# Angle-closure glaucoma associated with vitreous prolapse after neodymium-doped yttrium-aluminumgarnet laser posterior capsulotomy

**DOI:** 10.5935/0004-2749.2022-0058

**Published:** 2024-03-05

**Authors:** Alexis Galeno Matos, José de Paula Barbosa Neto, Carlos Philliph Pinheiro Cavalcante, Lucas Parente de Andrade, Jayter Silva Paula

**Affiliations:** 1 Fundação Leiria de Andrade, Fortaleza, CE, Brazil; 2 Universidade Federal de São Paulo, São Paulo, SP, Brazil; 3 Fundação de Ciência e Pesquisa Maria Ione Xerez Vasconcelos, Fortaleza, CE, Brazil; 4 Department of Ophthalmology, Otorhinolaryngology, and Head and Neck Surgery, Faculdade de Medicina de Ribeirão Preto, Universidade de São Paulo, Ribeirão Preto, SP, Brazil

**Keywords:** Glaucoma, Glaucoma angle closure, Cataract, Posterior capsulotomy, Laser, solid-state

## Abstract

Capsulotomy with neodymium-doped yttriumaluminum-garnet (Nd:YAG) laser is an effective treatment for posterior capsule opacification following cataract surgery. A wide opening of the posterior capsule associated with the ruptured anterior hyaloid can cause anterior chamber vitreous prolapse. Two patients who developed angle-closure glaucoma associated with vitreous prolapse following Nd:YAG laser posterior capsulotomy were successfully treated with antiglaucoma medication and peripheral iridotomies. Patient identification for potential risk factors and a careful postoperative follow-up are essential to avoid these serious complications.

## INTRODUCTION

Posterior capsule opacification (PCO) is caused by the proliferation of residual lens epithelial cells in the capsular bag, usually following cataract surgery. PCO incidence ranges from 3% to 50% in the first 5 years postoperatively^(1,2)^. Neodymium-doped yttrium-aluminumgarnet (Nd:YAG) laser is a global approach for posterior capsulotomy^(2)^.

A vitreous pupillary blockage is an uncommon cause of intraocular pressure (lOP) elevation after Nd:YAG laser capsulotomy, when a knuckle of vitreous insinuates around the intraocular lens (IOL) toward the anterior chamber^(3)^. Very few cases of angle-closure glaucoma secondary to vitreous prolapse after laser capsulotomy have been reported, and risk factor identification is essential to avoid serious complications^(4,5)^. This study aimed to investigate findings associated with acute angleclosure glaucoma in two patients presenting vitreous prolapse and pupillary block after laser capsulotomy.

## CASE REPORTS

### Case 1

A 79-year-old man, with no history of glaucoma but who reported phaco emulsification surgery 1 year earlier, was referred to an ophthalmology emergency department complaining of severe pain in the right eye (OD) associated with a significantly decreased visual acuity (20/400). He underwent an uneventful Nd:YAG laser capsulotomy 1 month before. Slit-lamp examination revealed a shallow anterior chamber with 360° iriscornea contact, corneal edema, and IOP of 58 mmHg in OD. Gonioscopy confirmed a 360° angle closure.

**Figure 1:**
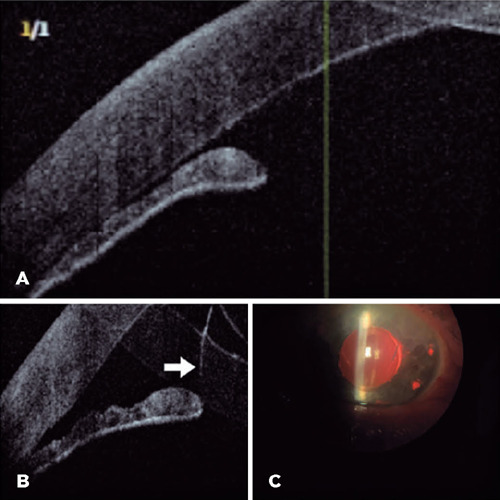
Anterior segment OCT image (A) of the frst patient showing the angle closure with anterior iris bulging. A vitreous prolapse insinuating around the IOL toward the anterior chamber. Anterior segment OCT (B) and slit-lamp images (C) of the frst patient showing the anterior chamber angle opening after peripheral iridotomies. The white arrow indicates the vitreous in the anterior chamber (B), and two iridotomies were observed in a wider anterior chamber (C).

The patient was treated with a combination of oral acetazolamide (250 mg) four times a day (QID) and a fixed topical combination of timolol maleate at 0.5% and brimonidine tartrate at 2% twice daily and topical prednisolone acetate at 1% six times a day. A peripheral iridotomy was performed later on the same day. The anterior chamber presented wilder with no iris-cornea contact 2 days following the procedure, and IOP was 14 mmHg, with excellent control for up to 2 months. Optical coherence tomography (OCT) examination confirmed angle opening after peripheral iridotomy, as well as vitreous in the anterior chamber (Figure 1).

### Case 2

A 58-year-old man was admitted to the ophthalmology emergency department with a sudden, painful de crease in visual acuity (20/200) in the left eye (OS). He had no history of glaucoma and underwent phacoemulsification surgery in both eyes 60 days before the onset of symptoms. Afterward, the patient underwent capsulotomy with Nd:YAG laser in the OS 30 days following cataract surgery. Slit-lamp examination displayed a shallow anterior chamber, corneal edema, and the presence of vitreous between the IOL and the iris, with posterior IOL displacement. IOP was 56 mmHg in OS, and gonioscopy confirmed a 360° angle closure.

**Figure 2:**
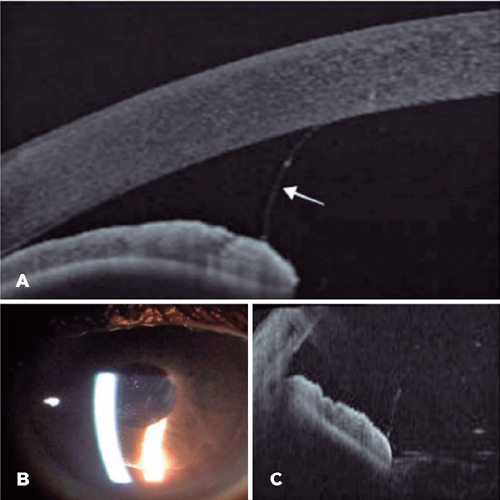
Anterior segment OCT image (A) of the second patient showing the aspect of the anterior chamber before iridotomy with vitreous prolapse (white arrow). Slit-lamp photography (B) and OCT images (C) of the second patient showing a wider anterior chamber, with vitreous observed in the pupillary region after the laser procedure. Posterior displacement of the iris along with a deeper anterior chamber after the peripheral laser iridotomy (C).

He was treated with oral acetazolamide (250 mg) QID, a fixed topical combination of timolol maleate at 0.5% and brimonidine tartrate at 2% twice daily, and prednisolone acetate at 1% QID. A peripheral Nd:YAG laser iridotomy was successfully completed on the same day. OCT examination after 1 month revealed a wider anterior chamber and prolapsed vitreous (Figure 2). The IOP was 14 mmHg in the following visits.

## DISCUSSION

Transient IOP increases are a common complication of the Nd:YAG laser capsulotomy^(1,6)^. IOP spikes are probably caused by obstruction of the trabecular meshwork by capsular remnants, inflammatory debris, and trabeculitis, which occurred as a multifactorial mechanism consequence^(3)^. Herein, we presented cases of acute angle-closure glaucoma after 30 days from laser capsulotomy in patients with pseudophakia with different postoperative periods. To the best of our knowledge, these cases displayed the longest period between the laser procedure and the onset of pupillary block. Unfortunately, we did not identify any ocular, systemic, or environmental causative factor for the late presentation of vitreous prolapse.

Similar findings have been observed between 1 day and 3 weeks after the laser procedure^(3,4)^. One group reported transient IOP elevations during the first 24 h, with 41% of the eyes presenting >30 mmHg and 14% presenting >40 mmHg^(7)^.

Laser energy would cause anterior vitreous disturbance, and high energy levels could rupture the anterior hyaloid causing immediate liquefaction, and its prolapse into the anterior chamber^(5)^. The posterior capsule opening could allow the aqueous humor misdirection, thereby creating an aqueous pocket pushing the IOL and iris forward^(3)^. Vitreous changes in the eyes of these two patients were not determined before the procedure.

Some studies have revealed no association between either the energy delivered or the capsulotomy size with increased IOP^(7)^. Patients with aphakia and pseudophakia who had either a large posterior capsule opening or great laser energy loads were more likely to have increased IOP due to vitreous prolapse^(6)^.

The IOL placement into the capsular bag is associated with a low probability of vitreous prolapse because the contact between the optical zone and the posterior capsule creates a mechanical barrier that would block any anterior movement of the vitreous body^(6,7)^. However, the risk of such movement could be higher in a larger laser aperture than in an IOL optical zone. Thus, the ideal capsulotomy-opening diameter should be recommended at 3.9–5.0 mm^(8)^. In addition, other authors have indicated either pupillary dilation for a few days to prevent pupillary block^(9)^ or a prophylactic iridotomy in patients with dense capsule opacity, previous glaucoma, or angular pathological conditions^(3,5)^. Further, the laser procedure could promote the capsular bag traction vector realignment, causing anterior capsule contraction and posterior IOL displacement toward the anterior vitreous^(10)^.

A previous study revealed that the use of an adequate energy load and a small capsule opening, even in cases presenting in-the-bag IOL, might have vitreous prolapse after laser capsulotomy. Further, the anterior hyaloid rupture because of the more posterior laser delivery would be the causative factor of vitreous prolapse^(4)^. Moreover, previous reports determined no association between ocular axial length and vitreous prolapse after laser capsulotomy. Unfortunately, the ocular axial length of the presented patients was not measured.

Although the patients’ IOLs were in-the-bag, capsular openings larger than the optical zone were observed. Potential damage to the anterior hyaloid associated with high energy loads delivered may have caused anterior prolapse. In both cases, the IOP reduction and the successful pupillary block reversal were achieved with a combination of peripheral iridotomy and the use of antiglaucoma and corticosteroid eye drops. This treatment restored the physiological aqueous humor flow, similar to a previous report^(5)^. Pilocarpine eye drops were not used because of the risk of pupillary block worsening after miosis.

Pupillary block due to vitreous prolapse is a rare event that can cause a fast IOP elevation. Thus, we recommend carefully assessing the risk factors presented in this discussion. Laser capsulotomy should not be considered within the first 4 months after cataract surgery, as well as large capsular openings and the use of high energy loads. In addition, antiglaucoma eye drops after the procedure and IOP measurements both within 4 h and 1 day after the procedure may help prevent and diagnose complications.
